# Responses of Bovine Innate Immunity to *Mycobacterium avium* subsp. *paratuberculosis* Infection Revealed by Changes in Gene Expression and Levels of MicroRNA

**DOI:** 10.1371/journal.pone.0164461

**Published:** 2016-10-19

**Authors:** Michela Malvisi, Fiorentina Palazzo, Nicola Morandi, Barbara Lazzari, John L. Williams, Giulio Pagnacco, Giulietta Minozzi

**Affiliations:** 1 Parco Tecnologico Padano, Lodi, Italy; 2 Department of Veterinary Medicine, University of Milan, Milan, Italy; 3 Faculty of Bioscience and Technology for Food, Agriculture and Environment, University of Teramo, Teramo, Italy; 4 Institute of Agricultural Biology and Biotechnology, National Research Council, Lodi, Italy; 5 School of Animal and Veterinary Sciences, University of Adelaide, Roseworthy, Australia; National Institute of Animal Biotechnology, INDIA

## Abstract

Paratuberculosis in cattle is a chronic granulomatous gastroenteritis caused by *Mycobacterium avium* subsp. *paratubercolosis* (MAP) which is endemic worldwide. In dairy herds, it is responsible for huge economic losses. However, current diagnostic methods do not detect subclinical infection making control of the disease difficult. The identification of MAP infected animals during the sub-clinical phase of infection would play a key role in preventing the dissemination of the pathogen and in reducing transmission. Gene expression and circulating microRNA (miRNA) signatures have been proposed as biomarkers of disease both in the human and veterinary medicine. In this paper, gene expression and related miRNA levels were investigated in cows positive for MAP, by ELISA and culture, in order to identify potential biomarkers to improve diagnosis of MAP infection. Three groups, each of 5 animals, were used to compare the results of gene expression from positive, exposed and negative cows. Overall 258 differentially expressed genes were identified between unexposed, exposed, but ELISA negative and positive groups which were involved in biological functions related to inflammatory response, lipid metabolism and small molecule biochemistry. Differentially expressed miRNA was also found among the three groups: 7 miRNAs were at a lower level and 2 at a higher level in positive animals vs unexposed animals, while 5 and 3 miRNAs were respectively reduced and increased in the exposed group compared to the unexposed group. Among the differentially expressed miRNAs 6 have been previously described as immune-response related and two were novel miRNAs. Analysis of the miRNA levels showed correlation with expression of their target genes, known to be involved in the immune process. This study suggests that miRNA expression is affected by MAP infection and play a key role in tuning the host response to infection. The miRNA and gene expression profiles may be biomarkers of infection and potential diagnostic of MAP infection earlier than the current ELISA based diagnostic tests.

## Background

*Mycobacterium avium* subspecies *paratuberculosis* (MAP) is the causal agent of paratuberculosis (paraTB) or Johne's disease in cattle, a chronic granulomatous gastroenteritis [1, 2]. ParaTB is endemic worldwide and occurs primarily in ruminants, including cattle, sheep, goats, and farmed deer. However, the disease has been reported in non-ruminants, such as wild rabbits [3], foxes and stoats [4] and in primates such as mandrills and macaques [5, 6].

Johne's disease causes substantial economic losses in dairy herds through lost productivity [7]. A link between MAP and Crohn's disease in humans has been suggested [8, 9], although the causal role of MAP has not yet been proven [10, 11] and the association remains controversial [11]. However, this possible risk to human health has increased interest in the disease and has made the need to improved diagnosis more pressing.

In cattle, the disease starts with the slow development of intestinal lesions in infected animals, a proportion of which become clinically ill two to six years after infection [12]. The disease progresses in four stages, which start with the silent phase, followed by subclinical, clinical and advanced phases [13]. Cows in the subclinical stages can be classified as low, moderate and high shedders, on the basis of the number bacteria that can be detected by fecal culture [14]. The subclinical stage is immunologically characterized by a protective Th1 immune response, and an elevated level of IFN-γ [15]. The progression of infection and appearance of clinical disease is associated with the shift from a Th1 to a non-protective Th2-mediated humoral response in the late subclinical phase [15].

The identification of infected animals at an early subclinical stage is critical to avoid transmission via the oral-fecal route and the dissemination of the pathogen. Infection mainly occurs in young calves, which are most susceptible, while adult cows are more resistant to infection [16]. The clinical phase is characterized by untreatable diarrhea, progressive weight loss, decreased milk production and ultimately death [17]. Currently infection is detected by an ELISA test to detect serum antibodies against MAP, or PCR of feces to detect the presence of the bacterium. However, antibodies are only present late in infection and detection of the bacterium by PCR has only moderate sensitivity when low shedders are tested [18, 19]. The fecal bacterial culture [20], does not identify subclinical cases [13, 21] and can give false positives when environmental MAP passes through uninfected animals or false negatives due to intermittent shedding.

There are no effective treatments for Johne's disease, and vaccine efficacy as protective tool in paratuberculosis prevention is still debatable [22]. Therefore, the detection and isolation of animals in the early stages of infection can play a key role in Johne's disease eradication. It is therefore important to develop a diagnostic test for animals during the early stages of infection, before they start shedding and spreading the disease.

Understanding host-pathogen interaction and disease responses has improved with the availability of high throughput—omics technologies. Studies have progressed from the analysis of candidate genes or loci associated with MAP susceptibility [23–25] to the investigation of the whole transcriptome. Expression microarrays have been used to analyze gene expression related to MAP infection in Holstein-Friesian orally-inoculated calves [26, 27]. Degradation of miRNA-targeted mRNA is now a well-known mechanism of post-transcriptional regulation of gene expression in plants and animals [28], although the levels of miRNAs and transcript abundance are poorly correlated [29]. Nevertheless, circulating miRNAs, contained in extracellular compartments such as plasma [30] or exosomes [31], have been identified as markers for human diseases, and are used in the diagnosis of some cancers [32], cardiovascular diseases [33], disorders of the immune system [34], neurodegenerative disease [35] and diabetes [36]. Significantly, miRNAs have been suggested as diagnostic markers of human tuberculosis infections [37]. In the veterinary field, specific miRNA patterns have been associated with viral diseases [38, 39] and gram-positive bacterial infections [40]. Although the mechanism leading the bacteria-induced miRNA expression changes has yet to be fully understood, both pathogenic and commensal bacteria have been shown to affect miRNA expression in the host [41]. Differentially expressed miRNAs have been suggested as diagnostic markers of MAP infection, but experimentally infected Holstein-Friesian calves did not show specific miRNA signatures, at least during the early latent period of MAP infection when diagnosis would be most useful [42]. In the present study, the mRNA and circulating miRNA expression were investigated in Holstein cattle positive and negative for MAP by ELISA to gain insights into gene regulatory networks related to Johne’s disease in clinical and preclinical phases of the disease.

## Results

### Differentially expressed genes and quantitative RT-PCR validation of the sequencing data

**Fig 1:**
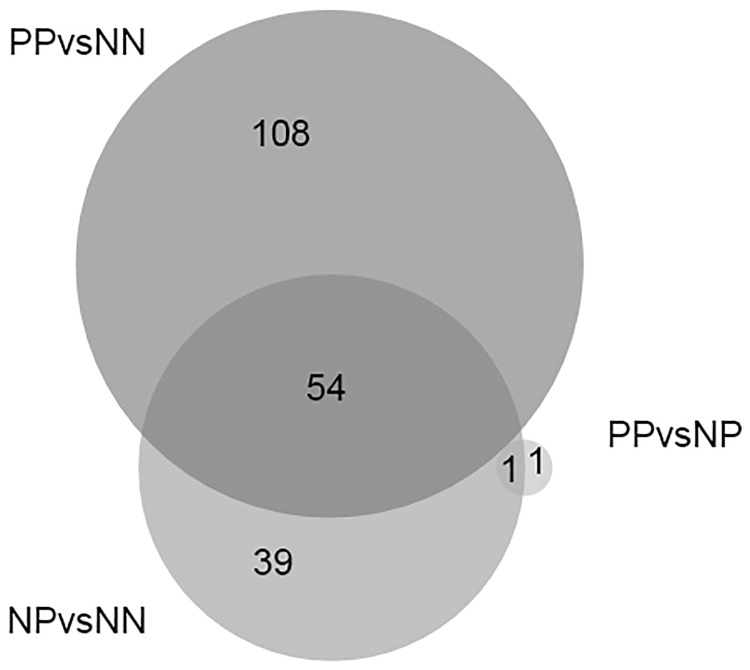
Venn diagram showing the number of common and specific differentially expressed genes for each comparison.

**Fig 2:**
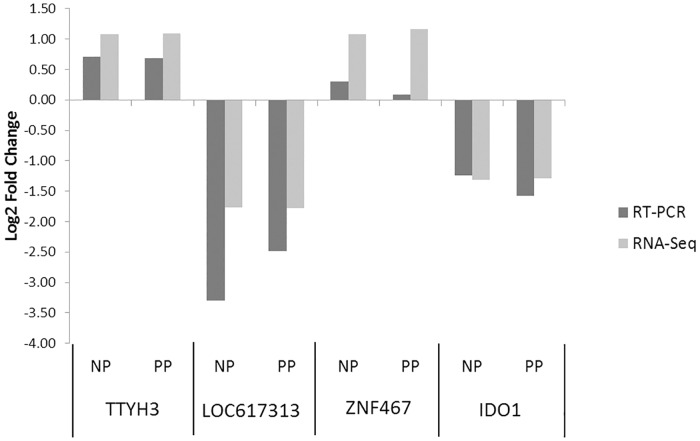
Validation of RNA-Seq data with qRT-PCR assays. Relative quantification was calculated with respect to the control group, after normalization with ACTB and GAPDH expression levels.

RNAseq data from whole blood of 5 infected (PP), 5 exposed (NP) and 5 negative (NN) animals identified 12,366 genes, 258 of which were differentially expressed in the three comparisons: 162 genes were differentially expressed (DE) comparing PP vs NN, 94 genes for NP vs NN and 2 genes for PP vs NP. In this study Log_2_ fold change above 1 or below -1 and 0.05 as false discovery rate (FDR) threshold were chosen to select DE genes. The complete lists of DE genes for the three comparisons are provided in supporting information files (S1, S2 and S3 Tables). Fifty-four DE genes were shared by PP vs NN and NP vs NN with the same trend of expression (Fig 1). All of animals were from the same breed and they have been carefully selected to be uniform for age, physiologic and health status but they differed for the ELISA test result. Thus the similarities found in the gene expression when the groups were compared with the control suggest that exposed animals may have been incubating disease in the preclinical stage. In addition, some animals of the NP group showed expression values similar to the PP group. A similar number of DE genes had increased vs decreased levels of expression in each comparison. The Log_2_ fold expression changes ranged from -5.0 to 3.6 for PP vs NN, -2.4 to 2.9 for NP vs NN and -2.7 to 1.7 in the PP vs NP comparisons. The Pearson correlation coefficient for RNA-Seq vs RT-qPCR data for *TTYH3*, *LOC617313*, *ZNF467* and *IDO1* which were DE in NP and PP group vs NN was 0.945 (p<0.01). (Fig 2).

### Differential expression of miRNAs

The miRNA-Seq data was used to identify known, and to predict novel miRNAs. Overall, 645 novel miRNAs were identified by the Mirdeep2 software [43].

Two samples (one PP and one NP) were excluded due to low read numbers and analysis for differential expression of miRNAs was conducted on the remaining 20 samples.

miRNAs with an FDR ≤0.05 were considered as differentially expressed. The comparison PP vs NN identified DE of 8 known and 1 novel miRNA. Among these, 7 had lower expression in the PP group compared with the NN group, with the novel miRNA 14_7917 having the greatest decrease (-6.54 Log_2_ FC), and 2 known miRNAs had higher expression in the PP group compared with the NN group (Table 1).

**Table 1:** Differentially expressed miRNAs in PP vs NN comparison.

Comparison	miRNA name	Chromosome	Log_2_FC*	PValue	FDR	Mature sequence
PP vs NN	bta-mir-19b	12	-1.38	<0.001	<0.001	UGUGCAAAUCCAUGCAAAACUGA
PP vs NN	bta-mir-19b-2	X	-1.40	<0.001	<0.001	UGUGCAAAUCCAUGCAAAACUGA
PP vs NN	bta-mir-1271	7	-0.88	<0.001	<0.001	CUUGGCACCUAGUAAGUACUCA
PP vs NN	bta-mir-100	15	-6.28	<0.001	0.009	AACCCGUAGAUCCGAACUUGUG
PP vs NN	bta-mir-301a	19	-2.38	<0.001	0.003	CAGUGCAAUAGUAUUGUCAAAGCAU
PP vs NN	bta-mir-32	8	-2.73	<0.001	0.008	UAUUGCACAUGACUAAGUUGCAU
PP vs NN	bta-mir-6517	10	1.25	<0.001	0.001	UCAGGGUCCGUGAGCUCCUCGGC
PP vs NN	bta-mir-7857	23	1.88	<0.001	<0.001	AUAGCCAGUUGGGGAAGAAUGC
PP vs NN	Novel:14_7917	14	-6.54	0.001	0.041	AGCAGCAGUGUACAGGGCUCUGA

* Log_2_ Fold Change in the positive group (PP) when compared with the unexposed group (NN).

Four miRNAs, bta-mir-19b, bta-mir-19b2, bta-mir-1271 and novel miRNA 14_7917, which showed a differential expression between PP and NN groups were also differentially expressed between NP vs NN groups with the same trend of expression. Other differentially expressed miRNAs between the NP and NN groups, which were not DE between PP and NN, were bta-mir-24-1, bta-mir-24-2, bta-mir-378c and Novel:27_25982 (Table 2).

**Table 2:** Differentially expressed miRNAs in NP vs NN comparison.

Comparison	miRNA name	Chromosome	Log_2_FC*	PValue	FDR	Mature sequence
NP vs NN	bta-mir-19b	12	-0.48	<0.001	0.001	UGUGCAAAUCCAUGCAAAACUGA
NP vs NN	bta-mir-19b-2	X	-0.51	<0.001	0.003	UGUGCAAAUCCAUGCAAAACUGA
NP vs NN	bta-mir-1271	7	-0.72	<0.001	<0.001	CUUGGCACCUAGUAAGUACUCA
NP vs NN	bta-mir-24-1	8	0.94	<0.001	<0.001	GUGCCUACUGAGCUGAUAUCAGU
NP vs NN	bta-mir-24-2	7	0.89	<0.001	<0.001	UGGCUCAGUUCAGCAGGAACAG
NP vs NN	bta-mir-378c	24	1.44	<0.001	0.017	ACUGGACUUGGAGUCAGAAGU
NP vs NN	Novel:14_7917	14	-6.54	<0.001	0.009	AGCAGCAGUGUACAGGGCUCUGA
NP vs NN	Novel:27_25982	27	-5.26	0.001	0.041	CCCGGUACUGAGCUGACCCGA

* Log_2_ Fold Change in the exposed group (NP) when compared with the unexposed group (NN)

Two miRNAs, bta-miR-19b and bta-miR-19b2, had lower levels of expression in the PP group vs the NP group with -0.9 Log_2_ Fold Change (Log_2_FC) (Table 3), while having reduced expression in PP vs NN and also showing reduced expression in the NP vs NN group, although to a lesser extent.

**Table 3:** miRNAs expression in PP compared with NP animals.

Comparison	miRNA name	Chromosome	Log_2_FC*	PValue	FDR	Mature sequence
PP vs NP	bta-mir-19b	12	-0.91	<0.001	0.002	UGUGCAAAUCCAUGCAAAACUGA
PP vs NP	bta-mir-19b-2	X	-0.89	<0.001	0.006	UGUGCAAAUCCAUGCAAAACUGA

* Log_2_ Fold Change in the positive group (PP) when compared with the exposed group (NP)

Most of the DE miRNAs have previously described in other species as having a role in immunity and inflammation. miRNAs involved in inflammation and immunological pathways and their target genes are summarized in Table 4.

**Table 4:** Differentially expressed miRNAs with inflammatory and immunological functions in other species.

DE miRNA	Species	Tissue	Disease	Expression	Target	Target function	Reference
mir-19b	Mouse	mir-17-92 deficient CD4+ T cells	\	Decreased	*PTEN*	Inhibition of PI3K-AKT-mTOR	[103]
	Mouse	mir-17-92 deficient Th1 cells	\	Decreased	*PTEN*	Inhibition of PI3K-AKT pathway	[104]
	Pig	PK15 cell line	PPV infection	Decreased	*DPP8*	Regulation of parvovirus immune response associated pathway	[105]
	Human	Serum	Tuberculosis	Decreased	n.d.	n.d.	[65]
	Human	Mucosal tissue	Crohn's disease	Decreased	n.d.	n.d.	[66]
mir-19b-2	Human	Sputum	Tuberculosis	Decreased	n.d.	Unknown	[62]
mir-100	Mouse	Lungs	Viral infection	Increased	n.d.	n.d.	[38]
	Shrimp	Hemocytes	Gram- infection	Increased	n.d.	n.d.	[60]
	Cow	Mammary epithelial cells	*S*. *uberis* infection	Decreased	n.d.	n.d.	[40]
mir-301a	Mouse	Lung	Viral infection	Decreased	n.d.	n.d.	[38]
	Mouse	TLR-triggered macrophages	\	Decreased	*NKRF*	Inhibition of NF-kB	[106]
mir-32	Cow	Alveolar macrophages	Bovine tuberculosis	Increased	n.d.	n.d.	[63]
	Human	CD4+ T cells	Tuberculosis	Decreased	n.d.		[61]
mir-24-2	Human	PBMC	Tuberculosis	Increased	n.d.		[67]
mir-378	Human	Serum	Tuberculosis	Increased	*MAPA1*	n.d.	[67]
	Human	Platelets	Ulcerative colitis	Increased	n.d.	n.d.	[68]
mir-1271	Human	Platelets	Ulcerative colitis	Increased	n.d.	n.d.	[68]

### Target prediction and validation of regulatory miRNAs

The correlation of expression between predicted targets and miRNA expression levels revealed that expression levels of 6 miRNAs, bta-mir-19b, bta-mir-19b-2, bta-mir-1271, bta-mir-32, bta-mir-7857 and the Novel:14_7917, were inversely correlated with the level of mRNA expression observed in the PP and NP groups compared to the NN group (Table 5).

**Table 5:** Correlation between miRNAs and predicted target genes.

miRNA name	miRNA Log_2_FC	Target score (miRDB)	Gene symbol	Gene description	Log_2_FC	FDR	Comparison
bta-mir-1271	-0.88	94	*TTYH3*	tweety family member 3	1.10	0.000	PP vs NN
bta-mir-19b	-1.38	94	*HIC1*	hypermethylated in cancer 1	1.17	0.035	PP vs NN
bta-mir-19b	-1.38	93	*TBC1D8*	TBC1 domain family, member 8 (with GRAM domain)	0.61	0.019	PP vs NN
bta-mir-19b	-1.38	91	*IMPDH1*	IMP (inosine 5'-monophosphate) dehydrogenase 1	0.56	0.017	PP vs NN
bta-mir-19b-2	-1.40	94	*HIC1*	hypermethylated in cancer 1	1.17	0.035	PP vs NN
bta-mir-19b-2	-1.40	93	*TBC1D8*	TBC1 domain family, member 8 (with GRAM domain)	0.61	0.019	PP vs NN
bta-mir-19b-2	-1.40	91	*IMPDH1*	IMP (inosine 5'-monophosphate) dehydrogenase 1	0.56	0.017	PP vs NN
bta-mir-32	-2.73	95	*FAM20C*	family with sequence similarity 20, member C	1.05	0.008	PP vs NN
bta-mir-32	-2.73	94	*MPP1*	membrane protein, palmitoylated 1, 55kDa	0.67	0.008	PP vs NN
bta-mir-7857	1.88	95	*ZBTB44*	zinc finger and BTB domain containing 44	-0.48	0.039	PP vs NN
Novel:14_7917	-6.54	91	*ARL2*	ADP-ribosylation factor-like 2	0.84	0.001	PP vs NN
Novel:14_7917	-6.54	99	*FAM78A*	family with sequence similarity 78, member A	0.49	0.041	PP vs NN
Novel:14_7917	-6.54	90	*AP2A1*	adaptor-related protein complex 2, alpha 1 subunit	0.48	0.023	PP vs NN
bta-mir-1271	-0.72	94	*TTYH3*	tweety family member 3	1.08	0.000	NP vs NN
bta-mir-19b	-0.48	90	*ZBTB4*	zinc finger and BTB domain containing 4	0.80	0.045	NP vs NN
bta-mir-19b-2	-0.51	90	*ZBTB4*	zinc finger and BTB domain containing 4	0.80	0.045	NP vs NN

Target genes with significant FDR and opposite pattern of expression from miRNA.

Bta-mir-19b and bta-mir-19b-2 share the same targets and therefore overall 12 genes were identified which showed expression consistent with being targets of miRNAs with differential level of expression, 10 of which were in the PP group and 2 in NP group compared with the NN group. Bta-mir-19b and bta-mir-19b2 were differentially expressed in both positive and exposed groups, although apparently affecting the expression of different target genes in the two groups. *HIC1*, *TBC1D8* and *IMPDH1* had the same sequence motif and were targeted by both bta-mir-19b and bta-mir-19b-2 in the PP group, while *ZBTB4* was targeted by bta-mir-19b and bta-mir-19b-2 in the NP group. None of the other differentially expressed genes were targets of more than one miRNA. bta-mir-1271 had significantly lower levels in both PP and NP groups compared to the NN group. *TTYH3*, which is a target for mir-1271, had a higher level of expression in PP and NP vs NN.

A novel miRNA, Novel:14_7917, showed a lower expression in PP vs NN animals. Putative targets of this miRNA (*FAM78A*, *ARL2* and *AP2A1*) showed higher levels of expression in the PP vs NN animals.

### Ingenuity Pathway Analysis

From the 258 DE genes among the three comparisons 174 could be mapped in the Ingenuity Knowledge Base database [44], 119 in PP vs NN animals and 55 in the NP vs NN animals.

The Ingenuity pathway analysis (IPA) of DE genes from the PP vs NN comparison identified biological functions related to inflammatory disease (27 molecules), inflammatory response (36 molecules), cellular movement (37 molecules), lipid metabolism (17 molecules), small molecules biochemistry (29 molecules), cell-to-cell signaling and interaction (31 molecules), and immune cell trafficking (27 molecules). In particular, 16 DE genes were found to be involved in inflammatory response, 7 in lipid metabolism and 12 in small molecule biochemistry associated functions.

IPA identified *“Eicosanoid Signaling”* and *“Leukotriene Biosynthesis”* as the top canonical pathways in both PP vs NN and NP vs NN comparisons (Table 6). In addition, the analysis of DE genes between PP and NN groups were associated with *“G-Protein Coupled Receptor Signaling”* and *“Granulocyte Adhesion and Diapedesis”* canonical pathways, while DE genes from the NP vs NN comparison were enriched *“EIF2 Signaling”* and *“Tryptophan Degradation to 2-amino-3-carboxymuconate Semialdheyde”*.

**Table 6:** Top canonical pathways associated with DE genes in PP vs NN and NP vs NN comparisons.

Ingenuity Canonical Pathways	Molecules	DE gene set
Eicosanoid Signaling	*ALOX15*, *LTC4S*, *ALOX5*, *CYSLTR2*	PP vs NN
Leukotriene Biosynthesis	*LTC4S*, *ALOX5*	PP vs NN
G-Protein Coupled Receptor Signaling	*P2RY14*, *ADORA3*, *CHRM3*, *ADRB3*, *PRKCG*	PP vs NN
Granulocyte Adhesion and Diapedesis	*VCAM1*, *IL1RL1*, *MMP14*, *HRH4*	PP vs NN
Leukotriene Biosynthesis	*LTA4H*, *LTC4S*	NP vs NN
EIF2 Signaling	*RPS15*, *RPS23*, *RPL26*, *RPL37*	NP vs NN
Eicosanoid Signaling	*LTA4H*, *LTC4S*	NP vs NN
Tryptophan Degradation to 2-amino-3-carboxymuconate Semialdehyde	*IDO1*	NP vs NN

Analysis of the IPA knowledge database for upstream regulators of the DE genes identified 3 significant predicted regulators that differed between PP and NN animals, namely *LEPR* (z- score = 2, activated), IL-12 complex (z-score = -2.410, inhibited) and IL-2 (z-score = -2.950, inhibited). In the NP vs NN comparison one upstream regulator gene, *MYCN*, was predicted to be activated (z-score 2.219).

### Functions and networks associated with DE genes in MAP infected or exposed dairy cows

**Fig 3:**
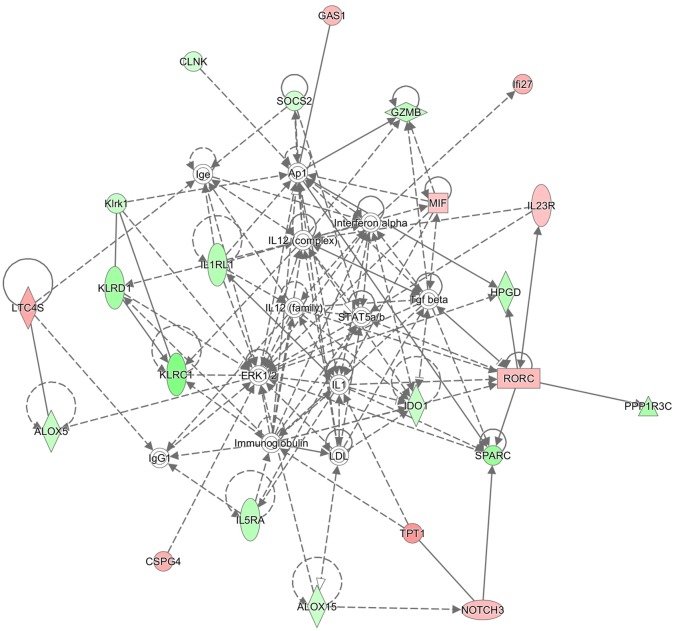
The most relevant network associated with DE genes of the PP vs NN comparison. Up-regulated (pink) and down-regulated (green) genes involved in lipid metabolism, small molecules biochemistry and Inflammatory response functions. Different shapes indicate different function of gene products: squares for cytokines, horizontal rectangles for ligand-dependent nuclear receptor, vertical rectangles for G-protein coupled receptors, vertical rhombus for enzymes, horizontal rhombus for peptidase, triangles for phosphatase, inverted triangle for kinase, vertical ovals for transmembrane receptors, horizontal ovals for transcription regulators, trapezius for transporters, double circles for complexes and circles for others.

**Fig 4:**
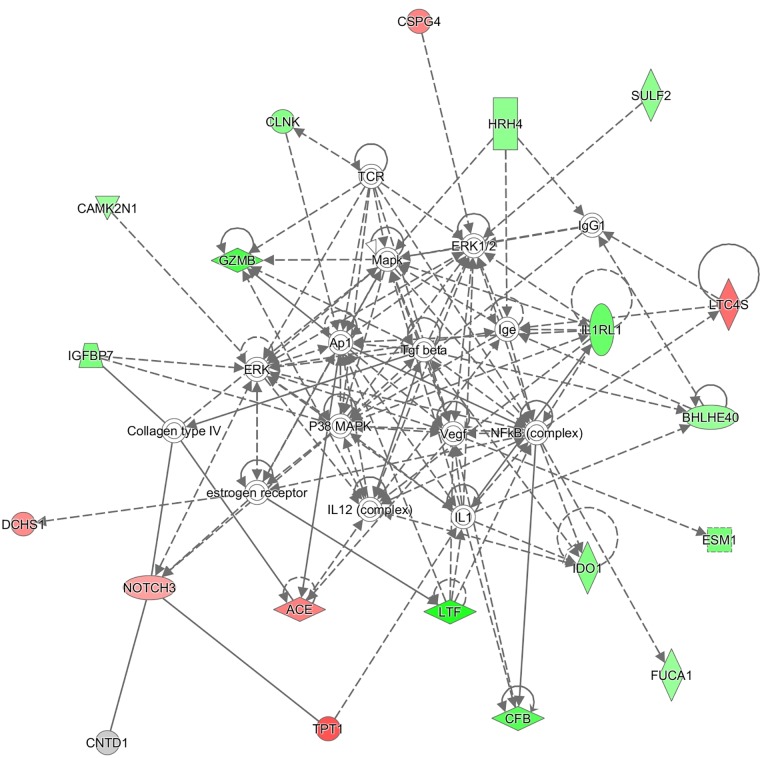
The most relevant network associated with DE genes of NP vs NN comparison. Up-regulated (pink) and down-regulated (green) genes involved in lipid metabolism, small molecules biochemistry and Inflammatory response functions. Different shapes indicate different function of gene products: squares for cytokines, horizontal rectangles for ligand-dependent nuclear receptor, vertical rectangles for G-protein coupled receptors, vertical rhombus for enzymes, horizontal rhombus for peptidase, triangles for phosphatase, inverted triangle for kinase, vertical ovals for transmembrane receptors, horizontal ovals for transcription regulators, trapezius for transporters, double circles for complexes and circles for others.

The DE genes observed in PP or NP cows versus NN, unexposed controls, were investigated for involvement in immunity-related functions. IPA analysis of gene expression profiles revealed networks associated with immune response and lipid metabolism. Gene networks defined for DE genes in the PP vs NN comparison were: “Lipid Metabolism, Small Molecule Biochemistry, Inflammatory Response” (score 45, focus molecules 22), “Inflammatory Disease, Respiratory Disease, Dermatological Diseases and conditions” (score 45, focus molecules 22), “Cell-To-Cell Signaling and Interaction, Cellular Growth and Proliferation, Hematological System Development and Function” (score 26, focus molecules 15), and “Cell Cycle, Digestive System Development and function, Cancer” (score 23, focus molecules 13). DE genes detected in the NP vs NN comparison were associated with several networks having a role in: “Inflammatory Response, Cellular Movement, Respiratory Disease” (score 46, focus molecules 20), “Cell Cycle, Nervous System Development and Function, Lipid Metabolism” (score 24, focus molecules 12), “Cell Signaling, Molecular Transport, Vitamin and Mineral Metabolism” (score 22, focus molecules 11) and “Cancer, Organismal Injury and Abnormalities, Molecular Transport” (score 21, focus molecules 11). The relationships, direct and indirect among the DE genes of PP vs NN and NP vs NN comparisons, are shown in Figs 3 and 4 respectively.

The representative network for the DE genes in the PP vs NN groups showed the relationships among the DE genes, as well as the predicted upstream and downstream effects of activation or inhibition molecules. The network includes *ALOX5* (arachidonate 5-lipoxygenase), *ALOX15* (arachidonate 15-lipoxygenase), *IDO1* (indoleamine 2,3-dioxygenase 1), *IL1RL1* (IL-1 receptor-like 1), *IL5RA* (IL-5 receptor, alpha), *SOCS2* (suppressor of cytokine signaling 2) among the down-regulated genes, while *IL23R* (IL-23 receptor) is one of the up-regulated genes in the network. From the IPA pathway analysis of genes associated with inflammatory response, genes with lower expression in the NP vs NN group, included *BHLHE40*, *CAMK2N1*, *CFB*, *CLNK*, *ESM1*, *FUCA1*, *GZMB*, *HRH4*, *IDO1*, *IGFBP7*, *IL1RL1*, *RTF* and *SULF2*, while *ACE*, *CSPG4*, *DCHS1*, *LTC4S*, *NOTCH3*, *TPT1* showed increased expression.

## Discussion

Early diagnosis of MAP is a big challenge for the control and the eradication of MAP from infected herds. The current study integrated the analysis of miRNA and mRNA expression data in order to relate gene expression with disease progression in cattle. Naturally infected adult cows, healthy cows from an infected herd and unexposed cows were compared to assess changes in the transcriptome associated with MAP infection, which may be useful for early diagnosis.

The analysis showed that expression of key inflammation and immune-related genes *ALOX5* and *ALOX15* was reduced in the PP group when compared with the NN group (S1 Table). The reduced expression of these genes has previously been described in calves 6 months after experimental inoculation with MAP [27]. The production of the enzyme 15-lipoxygenase, encoded by the *ALOX15* gene, is induced by IL-4 in monocytes and macrophages during Th2 type response [45]. Mice deficient in *12/15-LOX* have increased susceptibility to chronic toxoplasmosis which is associated with reduced production of IL-12 and gamma interferon (INF-γ) [46]. Thus, reduced expression of this gene is associated with a modified immune inflammatory response and may be related to the long term persistence of MAP in infected animals. IPA includes *ALOX5* and *ALOX15* within a gene network enriched for molecules associated with inflammatory response. This network encompasses several genes found to have lower expression in the PP vs NN group, including *IL1RL1*, *IDO1*, *IL5RA*, *SOCS2*, while *IL23R* showed increased expression (Fig 3).

*IL23R* and *SOCS2* are involved in cell-mediated immune response. *SOCS2* is a regulator of Th2 response, and homozygous knockout mice have a higher Th2 immune response after helminth antigen stimulation compared with wild-type mice [47]. IL-23 is produced by macrophages and dendritic cells and is a regulator of inflammation and of Th1 response. *IL-23* is over expressed in the ileal mucosa of sheep with paratuberculosis [48]. These findings suggest that the enhanced expression of *IL-23R* in infected animals, stimulates the Th1 immune response. However, the reduced *SOCS2* expression, may inhibit responses that are unfavourable to the intracellular pathogens survival, and promote a Th2 response.

*IDO1* expression was lower in the PP than NN as well as in the NP vs NN group (S2 Table) and has been found to have increased expression in the ileum, jejunum and ileum draining lymph nodes of MAP infected sheep [49]. IDO expression is known to be induced by IFN-γ [49, 50] and lower expression may be associated with the shift towards a Th2 response, typical of the JD progression. The shift from Th1 to Th2 response would also be stimulated by the decrease of *SOCS2* expression in the PP group. IPA includes *IDO1* within a gene network enriched for genes associated with inflammatory response in both PP and NP group. This network included additional genes found to have lower expression in the NP vs NN group, such as *IL1RL1* (Fig 4).

The reduced expression level of *IL1RL1* in the PP vs NN group was accompanied by an increase in *IL23R*. However, in NP vs NN the expression of *IL1RL1* was reduced although the expression of *IL23R* was unchanged. Increased *IL1RL1* expression is associated with the activation of Th2 response [51], while reduced *IL1RL1* may maintain the Th1 response. A Th1 response is required for pathogen elimination and *IL23R* up-regulation occurred only in the positive group indicate a further effort for the pathogen clearance.

### Predicted upstream regulators of DE genes in MAP infected or exposed dairy cows compared to control group

The analysis of the regulators of DE genes from the PP vs NN comparison revealed the LEPR trans-membrane receptor was activated in the PP group, while the IL-12 complex and IL-2 regulators were predicted to be inhibited. *GZMB*, *IDO1*, *KLRC1*, *KLRD1* and *SOCS2* are known to be regulated by the IL-12 complex and had reduced expression in MAP infected animals. In contrast, the *MIF* gene, which codes for the multifunctional cytokine ‘macrophage migration inhibitory factor’, and which is known to be down-regulated by the IL-12 complex [52], showed higher expression in the PP vs NN group. IL-12 cytokine has been shown to induce INF-γ production in primary CD4+T cells [53, 54], which is essential for the activation of macrophages and pathogen clearance. Furthermore, it is known that there is a synergic effect of IL-12 and IL-18 in the enhancement of the NK cells cytotoxicity and Th1 cell differentiation [55]. CD4+ Th1 cells are thought to play a key role in the protection against mycobacteria by production of IFN-γ, which activates infected macrophages to kill the intracellular bacteria [56]. The benefit of IFN-γ as anti-mycobacterial has been demonstrated in patients with IL-12 deficiency [57]. The reduction of IL-12 expression observed in the PP group may have led to a reduction of immune response against MAP. These observations support the hypothesis that the regulation of the host IL-12 response may be a way for MAP to prolong its survival in monocyte cells. Similarly, inhibition of IL-2 regulators was observed in the PP vs NN group. The decline in T-cell function in advanced mycobacterial infections in mice has been associated with impaired production of IL-2 leading to a decline in proliferation of activated T-cells and the loss of IFN-γ [58]. Taken together, these observations suggest the suppression of macrophages immune response and the suppression of T-cells recognition and effector functions, may contribute to the prolonged survival of MAP in macrophages. Indeed, the association of mycobacterial infections with the decline in the cellular immune function of the host is well known [59].

Expression levels of *RPS23*, *RPS15*, *RPL37*, *RPL26* and *IGFBP7* in NP animals predicted the activation of transcriptional regulator *MYCN*. *MYC* prevents the activation of *Nramp1* in mouse macrophages through the competition with IRF-8, leading to an impaired killing of intra-phagosomal pathogens [60]. Activation of *MYCN* in the NP group, which were clinically healthy and in which MAP was not detectable but which had been exposed to pathogen and may have bene incubating disease, indicates that MAP is able to prevent the activation of macrophages in order to ensure its intra-cellular survival.

### miRNAs as potential biomarkers of MAP infection

Some of the miRNAs found to be DE between the NN and PP groups have been described as having a role in immune function and inflammation following bacterial or viral infection. Expression of mir-100 was at lower levels in the PP vs the NN group in the present study (Table 1), although increased levels of mir-100 have been described in mouse lung after viral infection [38] and even in hemocytes from gram-negative infected shrimps [61]. However, reduced mir-100 expression has been observed in a bovine mammary epithelial cell line after the infection with *streptococcus uberis* [40], indicating that the miRNA response is associated with gram-positive bacterial infection. Mir-301a is an activator of NF-kB [62] and was under-expressed in PP vs NN groups. A decreased expression of mir-301a has been associated with both viral [38] and mycobacterial infection [63]. Levels of mir-32 were lower in PP vs NN groups in the present study while previous studies on bovine and human pulmonary tuberculosis reported the overexpression of mir-32 in infected alveolar macrophages [64] and CD4+ T cells [65]. Inconsistencies in miRNA expression between the mixed cell populations found in PBMC versus single cell types, specifically CD4+ T cells, has been observed [65], as well as in miRNA signatures from different biological fluids [63]. These differences may reflect different cell functions, or changes in expression levels only in cells that are actively infected with MAP.

Among differentially expressed miRNAs between the PP and NN groups bta-mir-19b, bta-mir-19b-2, bta-mir-1271 and Novel:14_7917 have previously been found to be involved in human tuberculosis and in inflammatory bowel disease. Interestingly, reduced expression of mir-19b and mir-19b-2 has been observed respectively in serum [66] and sputum [63] of tuberculosis affected human vs healthy patients. Furthermore, mir-19b was reported to have low levels of expression in mucosal tissue of Crohn’s affected patients when compared to healthy controls [67]. In the present study exposed animals (NP group) had an increased level of expression of bta-mir-24-1, bta-mir-24-2, bta-mir-378c compared with the NN group (Table 2) and higher variability in the NP group compared to the other two groups. Increased expression of mir-24-2 [68] and mir-378 [69], has been reported in serum of human patients with pulmonary tuberculosis and mir-378 increased expression was also reported in platelets of ulcerative colitis human patients [70] when compared to the healthy controls. In the present study, while mir-24-1, mir-24-2 and mir-378c levels were the same in NN and PP groups of cows, increased levels were observed in the exposed (NP) group. Exposed animals were from a herd with high MAP infection rate and most likely were in contact with the pathogen. However, the infection status of each NP individual is not known and it is possible that animals were infected at different stages of disease progression. This would explain the high variability of the expression levels observed in the exposed group compared with the other two groups for bta-mir-19b, bta-mir-19b2, bta-mir-301a and bta-mir-32 that were all differentially expressed between the PP vs NN groups. It should also be noted that not all the animals infected with MAP progress to clinical disease, and it is unclear whether some animals are able to develop an immune response that controls or eliminates the pathogen [71]. The differentially expressed miRNAs observed here may be involved in regulating early immune response and may play a role in controlling disease progression.

### Target genes and their role in immune function

The expression levels of genes potentially targeted by the differentially expressed miRNAs related to immune function was investigated and the effect of such regulation is summarized in S4 Table. *HIC1*, *TBC1D8* and *IMPDH1* were over expressed in the PP group in comparison with the NN control groups and had the same target sequence motif for both bta-mir-19b and bta-mir-19b-2, which had lower expression in the PP group. None of the other differentially expressed genes was targeted by more than one miRNA (Table 5).

Interestingly, *ZBTB44* (under expressed in the PP group) and *ZBTB4* (over expressed in the NP group) belonged to the same zinc finger and BTB domain-containing family of the BTB-ZF and POK (POZ and Krüppel) families [72]. miRNAs belonging to the human miR-17-92 cluster, including mir-19b, which had lower expression in NP vs NN, have been described as targeting *ZBTB4* [73] which indeed had higher expression levels in the NP group. Experimentally infected human macrophages showed lower levels of *ZBTB4* in phagosomes infected with an attenuated *Mycobacterium avium* subsp. *hominissuis* strain, compared with macrophage infected with the more virulent wild-type mycobacteria [74]. The attenuated strain, unlike the wild-type strain, was not able to replicate in macrophages or prevent phagosome-lysosome fusion. In the present study, the high expression of *ZBTB4* in the NP cows may suggest a response of macrophages to the pathogen invasion. *ZBTB44*, which had lower expression in PP vs NN groups, carries the target motif for bta-mir-7857, which had higher levels in PP vs NN. These observations indicate that levels of key response genes in the macrophage are being regulated by RNA interference mechanisms and not changes in transcription *per se*.

*TTYH3*, which is targeted by bta-mir-1271, had higher expression in both PP and NP groups compared to the unexposed NN group. *TTYH3* codes for a member of the tweety family proteins, which are calcium-dependent chloride channels that allow the passage of the chloride anion through the cell membrane for the stabilization of the membrane potential, transport, and cell volume regulation. Anionic channels play a key role in the maintenance of peripheral blood mononuclear cell functions, in particular those connected with innate immune response: NADPH oxidase activity, phagocytosis, and trans-endothelial migration [75]. Increased expression of *TTYH2*, a paralogue of *TTYH3*, has been observed in the whole blood of Holstein cows infected with *Mycobacterium avium* subsp. *paratuberculosis* [76]. The results presented here confirm the increased expression levels of this gene family in PP animals compared with the NN group, suggesting the involvement of *TTYH3* in the response to MAP challenge.

The serologically positive (PP) animals were from the same herd as the exposed serologically negative (NP) animals of the same age and sex. Patterns of miRNA and mRNA expression observed between the two groups suggests that the status was different, although expression of bta-mir-19b and bta-mir-19b2 differed in both NP and PP groups in comparison with the NN control group (Table 3), suggesting that the exposed group was responding, possibly to latent MAP infection. Interestingly different target genes of the miRNAs were differentially expressed in the PP vs NN or in the NP vs NN groups. One of the genes targeted by both bta-mir-19b and bta-mir-19b2 is *HIC1* (Hypermethylated In Cancer 1), which showed an increased expression only in the PP group which is consistent with the decrease in the miRNAs levels. *HIC1* controls the expression of toll-like receptor 2, which enhances the NF-kB related axis [77] and pro-inflammatory cytokine secretion. NF-kB activation, and an enhanced production of inflammatory mediators, has been observed in macrophages and other cells types after *Mycobacterium tuberculosis* infection, as well as in intestinal epithelial cells after mycobacteria challenge [78]. This suggests that the host immune response to the infection is being carefully regulated in order to avoid the uncontrolled release of inflammatory mediators.

Mir-19a, has been shown to target *IMPDH1*, decreasing the related protein expression level in breast cancer cells [79]. The target prediction analysis carried out here also suggests that bta-mir-19b and bta-mir-19b-2, which had lower levels in PP vs NN animals, target *IMPDH1*, which is consistent with the increased *IMPDH1* level in PP vs NN groups. Interestingly, although the level of mir-19 was also lower in the NP vs NN animals, the expression of *IMPDH1* was not appreciably higher in NP vs NN groups. *IMPDH1* codes for an inosine 5′-monophosphate dehydrogenase enzyme, which is essential for purine synthesis in lymphocytes, catalyzing the conversion of IMP to GMP [80]. *IMPDH* inhibition has anti-proliferative effects in human monocytes *in vitro* [81], and is associated with reduced pro-inflammatory cytokines, nitric oxide and lactate dehydrogenase production in a murine macrophage cell line [82]. It has also been shown that *IMPDH* inhibitors can reduce IFN-γ mRNA levels after allogeneic stimulation in mice [83]. Thus *IMPDH* activity seems to be essential to the normal proliferation, pro-inflammatory functions and activation of macrophages. The higher expression levels detected in PP animals vs NN controls, suggest the activation of cellular immune response upon MAP infection.

The present study identified a novel miRNA, Novel:14_7917, the expression of which was lower in PP vs NN animals. Predicted targets for this miRNA included *FAM78A*, *ARL2* and *AP2A1*, which is consistent with the higher expression seen in PP vs NN. *AP2A1* and *ARL2* are involved in the movement of vesicles through the cellular membrane. *AP2A1* is involved in endocytosis [84–86], while *ARL2* plays a role in both the regulation of tubulin folding and microtubule remodeling [87], which is essential for phagosome maturation [88]. Thus, the enhanced expression of these genes observed in the PP group may be associated with increased intracellular traffic due to phagocytosis related to the uptake of MAP.

The transcriptome and miRNA analysis indicated that mechanisms of innate and acquired immunity were affected, which may influence the status of the infecting mycobacterium. These data suggest a balance between increased and suppressed immune mechanisms, reflecting opposing action of the host response to clear infection and the strategy used by the pathogen to ensure survival within the host cells. The miRNA profiles and levels of predicted gene targets were consistent, that is increased miRNA was associated with a decrease in target genes and vice versa, suggesting that expression of these genes was regulated at the level of RNA interference not at the level of transcription. The status of animals in the NP group was not known however the analysis suggested that animals in this group were responding to the pathogen and may have been at an earlier stage of infection. In particular, the NP group showed reduced levels of bta-mir-19b, bta-mir-19b-2 and bta-mir-1271 and an increased expression of target genes which was observed in the PP vs NN groups. This pattern could indicate that signs of infection can be detected prior to sero-conversion and hence prior to the current ELISA diagnostic test. However, more work will be necessary to confirm this and test the specificity of the response. In conclusion, examining the transcriptome and miRNA profiles can focus analyses on specific pathways, in this case identifying host pathogen interactions. This facilitated the identification of miRNA and gene candidates for diagnostic tools to detect animals infected with MAP at an early stage of disease than the current ELISA test.

## Methods

### Animal resource

Holstein dairy cows were selected from two herds, one MAP free (verified by ELISA and absence of clinical cases) and the other positive for MAP (verified by ELISA, fecal culture and presence of clinical cases). From the positive herd, 5 positive (PP) cows based on ELISA test results and 5 ELISA negative, potentially exposed (NP) animals were chosen. All animals were 4 to 5 years old, had a body condition score (BCS) of 3 and were at 170 to 190 days in milk (DIM) when sampled. Positive subjects were confirmed by fecal culture. Five negative non-exposed control animals (NN) were selected from the negative herd, matched for age, lactation, BCS and DIM with the positive and exposed animals. For the miRNA-Seq experiment, in addition to these animals, 7 negative cows from the same negative herd were added to enlarge the control group.

### Sample preparation, RNA extraction and quality control

Samples used were taken during obligatory routine animal sanitary controls by an authorized veterinarian. No ethical approval was required, in compliance with the European Directive 2010/63/UE and the Italian regulation D. Lgs n. 26/2014. Whole blood was collected into PAXgene^®^ Blood RNA Tubes (PreAnalytiX GmbH) and kept at room temperature for at least two hours before freezing at -20°C for 24 hours before being transferred at -80°C until processing, as recommended by the manufacturer. Total RNA was extracted using the PAXgene^®^ Blood miRNA Kit (PreAnalytiX GmbH) according to the manufacturer’s protocol. Total RNA was eluted in a final volume of 80 μL. RNA concentration was measured by NanoDrop^™^ 1000 spectrophotometer (Thermo Scientific) and RNA integrity was assessed with an Agilent 2200 TapeStation system (Agilent Technologies).

### RNA-Seq library preparation and sequencing

Libraries were prepared with the Illumina Truseq RNA sample prep kit (Illumina Inc., USA) following manufacture’s protocol and the size and yield evaluated using an Agilent TapeStation 2200. Libraries were then quantified with an ABI9700 qPCR instrument using the KAPA Library Quantification Kit in triplicate, according to the manufacture’s protocol (Kapa Biosystems, Woburn, MA, USA) and then normalized to 10 nM as recommended by Illumina for cluster generation on the Hiseq2000. Fifteen libraries were prepared and equimolar amounts of 5 samples were mixed before NaOH denaturation. Each of the pools was run in a lane of a Hiseq Flowcell.

The Illumina Truseq PE cluster kit v3 was used to generate clusters on the grafted Illumina Flowcell and the hybridized libraries were sequenced on a Hiseq2000 with a 100 cycles of paired-end sequencing module using the Truseq SBS kit v3 (Illumina Inc., USA).

### miRNA-Seq library preparation and sequencing

For each sample, 5 μl of RNA were used to prepare a library with the TruSeq SmallRNA kit (Illumina Inc., USA) following the manufacturer’s instructions. In order to minimize primer dimers formation, half of the TruSeq Small RNA sample reagents were used, followed by 11 PCR cycles of PCR to amplify the library. 10 μl of unique indexed libraries were pooled and DNA fragments from 140 to 160 bp (the length of miRNA inserts plus the 3′ and 5′ adaptors) were selected from a Pippin 3% Agarose Dye free Gel cassette (BluPippin, Sage Science, MA, USA), which were then recovered in 40 μL of Pippin elution buffer. Fragments were purified by Qiagen MinElute PCR Purification kit (Qiagen, CA, USA). The indexed libraries were quantified as described above. Two pools of 11 sample libraries were prepared and 10 μL of each pool at a final concentration of 2 nM were used in a lane for 50bp Single-Read sequencing for a total of 2 lanes of an Illumina HiSeq2000.

### RNA-Seq data analysis

Preliminary quality control of raw reads was carried out with FastQC software v0.11.2 [89]. Illumina raw sequences were trimmed using Trimmomatic [90] and PCR primers and Illumina adapter sequences were removed. Minimum base quality 15 over a 4 base sliding window was required, then only sequences longer than 36 nucleotides were included in the downstream analysis. Reads which successfully passed trimming were mapped against the *Bos taurus* UMD3.1.68 reference genome sequence, using STAR [91] aligner to obtain BAM alignment files. The BAM files were sorted and indexed using Samtools [92]. In order to quantify counts for each sample a list of genes and relative abundance of mapping reads were extracted using htseq-count [93]. These count files were used for downstream statistical analysis.

### miRNAseq data analysis

Trimming and quality control were performed as described for RNA-Seq analysis, with the difference that a 15 nucleotide minimum sequence length was required.

Reads which passed the quality control were used for novel small-RNA discovery using Mirdeep2 [43]. Both “cow” and “human” known small-RNAs (mature and precursors) were downloaded from MirBase [94] and used as support datasets to help the discovery process. Absolute positions of novel miRNAs on the bovine reference genome were retrieved by BLAST [95]. Counts of all the known and the novel miRNAs were used to quantify expression levels for each sample using Mirdeep2. This pipeline produced a list of small-RNA IDs and the relative abundance of mapping reads (counts) for each sample which was used in the downstream statistical analysis.

### Expression Analysis of mRNAs and miRNAs

Statistical analyses to compare mRNA and miRNA expression profiles were performed using the "R" statistical environment edgeR [96], vegan [97] and gplots [98] packages. Genes that had at least one count per million in at least 3 samples were included in the gene expression analysis. A general linear model was used in the edge R Package to generate lists of mRNAs and miRNAs with statistically significant different expression among the three comparisons: PP vs NN, NP vs NN and PP vs NP. Differentially expressed mRNAs and miRNAs were defined as having a False Discovery Rate (FDR) below 0.05.

### Real-time RT-qPCR for RNA-Seq validation

To validate the RNA-Seq gene expression data RT-qPCR was performed on 4 genes selected from those which were differentially expressed in both PP vs NN and NP vs NN comparisons and had a FDR<0.01. Three hundred ng of total RNA were reverse transcribed into cDNA using the QuantiTect Reverse Transcription Kit (Qiagen), RT-qPCR was performed using 5 ng cDNA, and 250 nM each primer in GoTaq qPCR Master Mix (Promega). The reaction mixtures were incubated in 384-wells plates at 95°C for 10 min, followed by 45 cycles of 95°C for 15 seconds and 62°C for 1 min using a CFX 384 Real-Time PCR Detection System (Bio-rad Laboratories, USA). All reactions were performed in triplicate and “no-template” controls were included. Following amplification, a melting curve analysis was performed to verify the specificity of the reactions. *GAPDH* and *ACTB* were chosen as the reference genes, as they were stably expressed in the RNAseq data, and assayed in the same samples to normalize the data. In order to determine the efficiency and the dynamic range of the reaction, for each primer pair a standard curve was constructed from triplicate assays for 4 dilutions from 30 ng to 0.03 ng of pooled cDNA samples. Primers for Real-time RT-qPCR are listed in Table 7 and were designed using Primer-BLAST (NCBI) or deduced by the literature. The relative expression level of each selected gene was calculated according to the Pfaffl method [99] and was reported as normalized fold expression relative to the control (NN cows). Log_2_ fold-change data of RT-qPCR and RNA-Seq analyses were compared to validate results. The Pearson correlation coefficient between the two analyses was calculated using IBM Statistical Package for Social Sciences software (SPSS, ver. 21; IMB SPSS Inc., Chicago, IL, USA) and differences were considered as statistically significant if the p-Value was <0.05.

**Table 7:** Primers used for RT-qPCR.

Gene symbol	Description	Sequence	Source
*GAPDH*	Glyceraldehyde-3-phosphate dehydrogenase	Forward 5'GGCGTGAACCACGAGAAGTATAA	[100]
		Reverse 5'CCCTCCACGATGCCAAAGT	
*ACTB*	Actin beta	Forward 5'GCAGGAGTACGATGAGTCCG	Primer BLAST
		Reverse 5'ATCCTGAGTCAAGCGCCAAA	
*TTYH3*	Tweety family member 3	Forward 5'ACATCTTGCAGTACTACCTGGC	Primer BLAST
		Reverse 5'GATTCACCTCTGTGCCGTTCAG	
*LOC617313*	Granzyme H	Forward 5'CCTTTCTTCTGCCTCCTGGG	Primer BLAST
		Reverse 5'GCCACACCTCTTCCAACTCT	
*ZNF467*	Zinc finger protein 467	Forward 5'AATTCCCTGCTCCTCACCG	Primer BLAST
		Reverse 5'TGTTCCCCAGGCTCACTTTG	
*IDO1*	Indoleamine 2,3-dioxygenase 1	Forward 5'GGGCATTCAGCACAGTATTGG	Primer BLAST
		Reverse 5'GGTGAGCTGGTGGCATGTA	

### Functional Analysis of gene expression data

The RNA-Seq differentially expressed (DE) gene lists obtained for each comparison (PP vs NN, NP vs NN, PP vs NP) were submitted to the Qiagen Ingenuity Pathway Analysis (IPA; Ingenuity Systems Inc., USA). The DE genes were filtered for an FDR≤0.05 and a fold change ≥1 or ≤-1 and were used as input in three separate data sets. Each gene was mapped to its corresponding gene object in the Ingenuity Knowledge Base. In the Core Analysis, IPA assigns functional information and biological relevance by analyzing RNA expression data in the context of known biological response and regulatory networks. In addition, canonical pathways, biological functions, and networks were investigated to identify major biological pathways associated with MAP infection. The upstream regulators were investigated based on an activation z-score, which was considered as significant when below -2 (inhibited) or above 2 (activated).

### Prediction of miRNA target genes and correlation with mRNA data

Due to the uncertainty associated with the prediction of miRNA target genes [40], two approaches were used. For known miRNAs only the gene targets identified by both methods were considered in the subsequent analysis. miRDB software [101] was used with default threshold settings for the initial prediction, based on miRNA-target matching the miRNA seed region and 3’ UTR mRNAs. The method was applied for both known and novel differentially expressed miRNAs and a threshold of 90 was set to select a panel of potential target genes. Predicted targets of known miRNAs were then analyzed with the TargetScan software v7.0 [102]. RNA-Seq expression levels were investigated for the genes that were shared between the two lists. Predicted gene targets of the novel miRNAs were obtained only from the miRDB prediction software, because target identification was done directly from the novel miRNA sequences. Targets which in RNA-Seq results had FDR <0.05 and opposite pattern of expression with respect to related miRNAs were considered as putatively affected by miRNA expression.

## Supporting Information

S1 Table Differentially expressed genes in the PP subjects when compared with the NN group. Genes with an FDR<0,05 and a Log_2_ fold change (Log_2_FC) <-1 or >1 were considered as differentially expressed.
(DOCX)

S2 Table Differentially expressed genes in the NP subjects when compared with the NN group. Genes with an FDR<0,05 and a Log_2_ fold change (Log_2_FC) <-1 or >1 were considered as differentially expressed.
(DOCX)

S3 Table Differentially expressed genes in the PP subjects when compared with the NP group. Genes with an FDR<0,05 and a Log_2_ fold change (Log_2_FC) <-1 or >1 were considered as differentially expressed.
(DOCX)

S4 Table Effects of gene regulation by miRNAs on the immune functions. (DOCX)
