# Supplementary material for: Responses of Bovine Innate Immunity to Mycobacterium avium subsp. paratuberculosis Infection Revealed by Changes in Gene Expression and Levels of MicroRNA
Source: PLoS One. 2016 Oct 19;11(10):e0164461. doi: 10.1371/journal.pone.0164461 (PMC5070780; doi:10.1371/journal.pone.0164461)
Supplement: S1 Table — Genes with an FDR<0,05 and a Log2 fold change (Log2FC) <-1 or >1 were considered as differentially expressed. (DOCX) [file pone.0164461.s001.docx]

**S1 Table**. D**ifferentially expressed genes in the positive subject when compared with the control group**. Genes with an FDR<0,05 and a Log2 fold change (LogFC) <-1 or >1 were considered as differentially expressed.

| **Sequence ID** | **Gene symbol** | **LogFC** | **FDR** |
| --- | --- | --- | --- |
| ENSBTAG00000002942 | SLC2A10 | 3.60 | 0.012620 |
| ENSBTAG00000039283 | novel gene | 3.38 | 0.000744 |
| ENSBTAG00000031849 | TMEM119 | 2.07 | 0.003607 |
| ENSBTAG00000033897 | LOC784762 | 1.97 | 0.012135 |
| ENSBTAG00000022275 | novel gene | 1.96 | 0.000044 |
| ENSBTAG00000039059 | GP1BB | 1.88 | 0.020010 |
| ENSBTAG00000047836 | novel gene | 1.87 | 0.000005 |
| ENSBTAG00000038913 | novel gene | 1.83 | 0.001073 |
| ENSBTAG00000004526 | TPT1 | 1.82 | 0.000021 |
| ENSBTAG00000011299 | novel gene | 1.75 | 0.000000 |
| ENSBTAG00000031453 | CCM2L | 1.73 | 0.018149 |
| ENSBTAG00000045673 | LOC613345 | 1.69 | 0.000468 |
| ENSBTAG00000003086 | RPLP2 | 1.63 | 0.001140 |
| ENSBTAG00000031795 | novel gene | 1.62 | 0.007453 |
| ENSBTAG00000014824 | MMP14 | 1.61 | 0.019666 |
| ENSBTAG00000040210 | novel gene | 1.59 | 0.000108 |
| ENSBTAG00000032702 | novel gene | 1.57 | 0.000005 |
| ENSBTAG00000046010 | BHLHA15 | 1.53 | 0.003726 |
| ENSBTAG00000029982 | MIR142 | 1.52 | 0.001973 |
| ENSBTAG00000009737 | LTC4S | 1.50 | 0.043235 |
| ENSBTAG00000047700 | novel gene | 1.50 | 0.007155 |
| ENSBTAG00000015040 | LOC100141215 | 1.49 | 0.008841 |
| ENSBTAG00000046245 | novel gene | 1.48 | 0.000005 |
| ENSBTAG00000012219 | CSPG4 | 1.42 | 0.022408 |
| ENSBTAG00000003152 | novel gene | 1.41 | 0.019666 |
| ENSBTAG00000047508 | ZNF580 | 1.36 | 0.000114 |
| ENSBTAG00000010060 | SLC43A3 | 1.35 | 0.027852 |
| ENSBTAG00000014666 | GP9 | 1.35 | 0.040135 |
| ENSBTAG00000017113 | ANKRD33 | 1.33 | 0.014076 |
| ENSBTAG00000046100 | novel gene | 1.32 | 0.000006 |
| ENSBTAG00000026489 | TNFRSF13C | 1.30 | 0.000052 |
| ENSBTAG00000017981 | ADRB3 | 1.29 | 0.014180 |
| ENSBTAG00000029996 | MIR27A | 1.29 | 0.033276 |
| ENSBTAG00000024280 | novel gene | 1.29 | 0.009768 |
| ENSBTAG00000014358 | EVA1B | 1.29 | 0.000799 |
| ENSBTAG00000021338 | OAF | 1.28 | 0.003116 |
| ENSBTAG00000008542 | SSPO | 1.27 | 0.020815 |
| ENSBTAG00000015405 | DCHS1 | 1.27 | 0.009681 |
| ENSBTAG00000046803 | GAS1 | 1.27 | 0.000744 |
| ENSBTAG00000007693 | SLC45A3 | 1.23 | 0.037975 |
| ENSBTAG00000019095 | CCR10 | 1.23 | 0.012081 |
| ENSBTAG00000024814 | B4GALNT4 | 1.23 | 0.023114 |
| ENSBTAG00000026501 | CYP2D14 | 1.22 | 0.000008 |
| ENSBTAG00000003923 | GPBAR1 | 1.21 | 0.000049 |
| ENSBTAG00000017405 | RORC | 1.21 | 0.003315 |
| ENSBTAG00000047926 | OVCA2 | 1.21 | 0.033083 |
| ENSBTAG00000023365 | LOC100297056 | 1.20 | 0.042990 |
| ENSBTAG00000043971 | NOTCH3 | 1.20 | 0.013604 |
| ENSBTAG00000018016 | NUPR1 | 1.19 | 0.038395 |
| ENSBTAG00000027386 | novel gene | 1.19 | 0.044830 |
| ENSBTAG00000020341 | MOV10L1 | 1.18 | 0.028551 |
| ENSBTAG00000027654 | EIF4EBP1 | 1.18 | 0.000009 |
| ENSBTAG00000011680 | PAPLN | 1.18 | 0.005871 |
| ENSBTAG00000018870 | OTOA | 1.18 | 0.043235 |
| ENSBTAG00000018920 | TRIM47 | 1.18 | 0.000154 |
| ENSBTAG00000025130 | HIC1 | 1.17 | 0.034922 |
| ENSBTAG00000038055 | KLF16 | 1.17 | 0.000021 |
| ENSBTAG00000019844 | ZNF467 | 1.16 | 0.000082 |
| ENSBTAG00000014705 | HES4 | 1.16 | 0.010625 |
| ENSBTAG00000019249 | HS3ST6 | 1.16 | 0.020815 |
| ENSBTAG00000047659 | novel gene | 1.15 | 0.005161 |
| ENSBTAG00000021602 | CTTNBP2NL | 1.12 | 0.018130 |
| ENSBTAG00000047538 | RIMBP3B | 1.11 | 0.042399 |
| ENSBTAG00000046140 | NPDC1 | 1.10 | 0.005763 |
| ENSBTAG00000015108 | USHBP1 | 1.10 | 0.014421 |
| ENSBTAG00000044158 | LDLRAD3 | 1.10 | 0.043561 |
| ENSBTAG00000005934 | TTYH3 | 1.10 | 0.000160 |
| ENSBTAG00000037757 | EBF4 | 1.09 | 0.017789 |
| ENSBTAG00000001824 | SLC2A6 | 1.09 | 0.000005 |
| ENSBTAG00000001814 | PLXND1 | 1.08 | 0.002506 |
| ENSBTAG00000030521 | IER5L | 1.06 | 0.000005 |
| ENSBTAG00000046486 | novel gene | 1.06 | 0.001277 |
| ENSBTAG00000007253 | TSPAN33 | 1.06 | 0.003840 |
| ENSBTAG00000003607 | FAM20C | 1.05 | 0.008322 |
| ENSBTAG00000014983 | IL23R | 1.05 | 0.043014 |
| ENSBTAG00000020532 | novel gene | 1.04 | 0.040725 |
| ENSBTAG00000000042 | PYCR1 | 1.04 | 0.011853 |
| ENSBTAG00000007375 | MIF | 1.03 | 0.000055 |
| ENSBTAG00000046111 | ZNF444 | 1.02 | 0.000189 |
| ENSBTAG00000000244 | NRXN2 | 1.02 | 0.005763 |
| ENSBTAG00000004093 | TUBB2B | 1.02 | 0.000618 |
| ENSBTAG00000021755 | novel gene | 1.01 | 0.000723 |
| ENSBTAG00000021013 | TUBB4A | 1.01 | 0.047503 |
| ENSBTAG00000034613 | MROH6 | 1.00 | 0.012189 |
| ENSBTAG00000016525 | ITGA1 | -1.00 | 0.024274 |
| ENSBTAG00000001457 | C20H5orf28 | -1.00 | 0.012135 |
| ENSBTAG00000046323 | novel gene | -1.02 | 0.035471 |
| ENSBTAG00000031388 | DYX1C1 | -1.02 | 0.040227 |
| ENSBTAG00000040051 | novel gene | -1.04 | 0.043039 |
| ENSBTAG00000007785 | PKD2L2 | -1.04 | 0.004015 |
| ENSBTAG00000011209 | SYT11 | -1.05 | 0.022173 |
| ENSBTAG00000007642 | ADORA3 | -1.05 | 0.048504 |
| ENSBTAG00000018864 | CENPU | -1.06 | 0.043559 |
| ENSBTAG00000017508 | CYSLTR2 | -1.06 | 0.010299 |
| ENSBTAG00000020710 | CENPQ | -1.07 | 0.004250 |
| ENSBTAG00000038340 | CLNK | -1.08 | 0.013924 |
| ENSBTAG00000015177 | PRSS23 | -1.09 | 0.005956 |
| ENSBTAG00000012007 | SOCS2 | -1.09 | 0.008841 |
| ENSBTAG00000006447 | ACSM3 | -1.09 | 0.022324 |
| ENSBTAG00000012907 | ODF2L | -1.12 | 0.001956 |
| ENSBTAG00000011227 | TRPC6 | -1.13 | 0.017826 |
| ENSBTAG00000019048 | novel gene | -1.14 | 0.016115 |
| ENSBTAG00000021361 | SAMD3 | -1.15 | 0.007383 |
| ENSBTAG00000019670 | SUCNR1 | -1.15 | 0.038069 |
| ENSBTAG00000016770 | GAPT | -1.15 | 0.040725 |
| ENSBTAG00000040281 | novel gene | -1.17 | 0.034633 |
| ENSBTAG00000018703 | OSTN | -1.17 | 0.012620 |
| ENSBTAG00000008059 | CHRM3 | -1.18 | 0.030701 |
| ENSBTAG00000011990 | ALOX15 | -1.19 | 0.045592 |
| ENSBTAG00000042616 | U6 | -1.20 | 0.044830 |
| ENSBTAG00000035129 | novel gene | -1.22 | 0.042822 |
| ENSBTAG00000001359 | B3GNT7 | -1.22 | 0.024187 |
| ENSBTAG00000019368 | IGFBP7 | -1.22 | 0.016306 |
| ENSBTAG00000038141 | novel gene | -1.23 | 0.012135 |
| ENSBTAG00000004917 | KLRK1 | -1.25 | 0.012083 |
| ENSBTAG00000047142 | LOC784249 | -1.26 | 0.018130 |
| ENSBTAG00000014750 | EPB41L4A | -1.26 | 0.040554 |
| ENSBTAG00000010728 | RAB44 | -1.27 | 0.025130 |
| ENSBTAG00000018206 | NIPAL2 | -1.28 | 0.001474 |
| ENSBTAG00000018446 | GCA | -1.28 | 0.001474 |
| ENSBTAG00000020602 | IDO1 | -1.29 | 0.003116 |
| ENSBTAG00000012991 | PRUNE2 | -1.30 | 0.003896 |
| ENSBTAG00000030426 | LOC100848575 | -1.33 | 0.048110 |
| ENSBTAG00000020319 | ALOX5 | -1.33 | 0.035004 |
| ENSBTAG00000017769 | RIBC1 | -1.34 | 0.034151 |
| ENSBTAG00000016548 | TEX12 | -1.34 | 0.040346 |
| ENSBTAG00000013550 | PRKCG | -1.35 | 0.022408 |
| ENSBTAG00000038981 | LOC518134 | -1.35 | 0.005734 |
| ENSBTAG00000045985 | novel gene | -1.35 | 0.019807 |
| ENSBTAG00000004221 | ESM1 | -1.46 | 0.005851 |
| ENSBTAG00000015483 | CCR8 | -1.47 | 0.000072 |
| ENSBTAG00000043985 | DACH1 | -1.47 | 0.034795 |
| ENSBTAG00000007109 | ASB2 | -1.47 | 0.004819 |
| ENSBTAG00000004263 | ATP6V0A4 | -1.48 | 0.000055 |
| ENSBTAG00000006643 | C6H4orf19 | -1.48 | 0.018149 |
| ENSBTAG00000010057 | GZMB | -1.50 | 0.034924 |
| ENSBTAG00000001125 | ADAMDEC1 | -1.51 | 0.049494 |
| ENSBTAG00000020467 | HRH4 | -1.52 | 0.000332 |
| ENSBTAG00000040367 | novel gene | -1.54 | 0.017595 |
| ENSBTAG00000000273 | IL5RA | -1.56 | 0.020815 |
| ENSBTAG00000009733 | FBP1 | -1.56 | 0.004919 |
| ENSBTAG00000047426 | LYPD6 | -1.59 | 0.029108 |
| ENSBTAG00000038080 | LOC508858 | -1.61 | 0.010271 |
| ENSBTAG00000025942 | HPGD | -1.62 | 0.019816 |
| ENSBTAG00000031265 | SVOPL | -1.63 | 0.006535 |
| ENSBTAG00000018571 | IL1RL1 | -1.66 | 0.001304 |
| ENSBTAG00000013055 | novel gene | -1.71 | 0.002045 |
| ENSBTAG00000046693 | novel gene | -1.74 | 0.003661 |
| ENSBTAG00000017448 | EFEMP1 | -1.75 | 0.011716 |
| ENSBTAG00000010828 | LOC617313 | -1.77 | 0.002929 |
| ENSBTAG00000038159 | novel gene | -1.81 | 0.009257 |
| ENSBTAG00000015118 | PNPLA1 | -1.82 | 0.048266 |
| ENSBTAG00000014831 | PPP1R3C | -1.86 | 0.048110 |
| ENSBTAG00000046668 | ANG2 | -1.93 | 0.028869 |
| ENSBTAG00000009252 | KLRA1 | -1.95 | 0.025779 |
| ENSBTAG00000046389 | KLRD1 | -2.02 | 0.000984 |
| ENSBTAG00000002092 | PI16 | -2.03 | 0.018130 |
| ENSBTAG00000014835 | SPARC | -2.28 | 0.045637 |
| ENSBTAG00000020990 | P2RY14 | -2.30 | 0.000052 |
| ENSBTAG00000003440 | novel gene | -2.35 | 0.005161 |
| ENSBTAG00000038843 | NKG2C | -2.76 | 0.004285 |
| ENSBTAG00000037539 | LOC534578 | -4.98 | 0.021329 |
