# Supplementary material for: Responses of Bovine Innate Immunity to Mycobacterium avium subsp. paratuberculosis Infection Revealed by Changes in Gene Expression and Levels of MicroRNA
Source: PLoS One. 2016 Oct 19;11(10):e0164461. doi: 10.1371/journal.pone.0164461 (PMC5070780; doi:10.1371/journal.pone.0164461)
Supplement: S2 Table — Genes with an FDR<0,05 and a Log2 fold change (Log2FC) <-1 or >1 were considered as differentially expressed. (DOCX) [file pone.0164461.s002.docx]

**S2 Table. Differentially expressed genes in the exposed subject when compared with the control group**. Genes with an FDR<0,05 and a Log2 fold change (Log2FC) <-1 or >1 were considered as differentially expressed.

| Sequence ID | Gene symbol | LogFC | FDR |
| --- | --- | --- | --- |
| ENSBTAG00000034449 | novel gene | 2.87 | 0.015412 |
| ENSBTAG00000043250 | 7SK | 2.76 | 0.025306 |
| ENSBTAG00000021807 | TAC3 | 2.52 | 0.039820 |
| ENSBTAG00000000820 | GNG11 | 2.48 | 0.020643 |
| ENSBTAG00000004526 | TPT1 | 2.12 | 0.000000 |
| ENSBTAG00000031849 | TMEM119 | 2.09 | 0.007448 |
| ENSBTAG00000011299 | novel gene | 2.09 | 0.000000 |
| ENSBTAG00000035654 | novel gene | 1.96 | 0.009059 |
| ENSBTAG00000047836 | novel gene | 1.88 | 0.000006 |
| ENSBTAG00000020368 | novel gene | 1.87 | 0.013113 |
| ENSBTAG00000046232 | novel gene | 1.83 | 0.009081 |
| ENSBTAG00000032702 | novel gene | 1.80 | 0.000000 |
| ENSBTAG00000009737 | LTC4S | 1.78 | 0.040494 |
| ENSBTAG00000046100 | novel gene | 1.76 | 0.000000 |
| ENSBTAG00000031795 | novel gene | 1.69 | 0.012842 |
| ENSBTAG00000004472 | DYNLT1 | 1.65 | 0.041565 |
| ENSBTAG00000005142 | RPL37 | 1.61 | 0.032244 |
| ENSBTAG00000040210 | novel gene | 1.61 | 0.000253 |
| ENSBTAG00000038913 | novel gene | 1.60 | 0.015667 |
| ENSBTAG00000013358 | RPS23 | 1.59 | 0.015028 |
| ENSBTAG00000022275 | novel gene | 1.57 | 0.004814 |
| ENSBTAG00000024950 | ACE | 1.57 | 0.017186 |
| ENSBTAG00000012344 | RPL26 | 1.56 | 0.000920 |
| ENSBTAG00000045673 | LOC613345 | 1.52 | 0.006652 |
| ENSBTAG00000012219 | CSPG4 | 1.52 | 0.039730 |
| ENSBTAG00000043582 | novel gene | 1.46 | 0.045940 |
| ENSBTAG00000047926 | OVCA2 | 1.45 | 0.025667 |
| ENSBTAG00000015405 | DCHS1 | 1.43 | 0.007710 |
| ENSBTAG00000033887 | RPL36A-HNRNPH2 | 1.40 | 0.006643 |
| ENSBTAG00000037945 | CASKIN1 | 1.37 | 0.018330 |
| ENSBTAG00000003086 | RPLP2 | 1.36 | 0.021136 |
| ENSBTAG00000025595 | novel gene | 1.33 | 0.013113 |
| ENSBTAG00000026489 | TNFRSF13C | 1.26 | 0.000217 |
| ENSBTAG00000046486 | novel gene | 1.23 | 0.000253 |
| ENSBTAG00000039470 | novel gene | 1.22 | 0.047849 |
| ENSBTAG00000046140 | NPDC1 | 1.21 | 0.005357 |
| ENSBTAG00000011680 | PAPLN | 1.19 | 0.013539 |
| ENSBTAG00000043971 | NOTCH3 | 1.17 | 0.040494 |
| ENSBTAG00000014358 | EVA1B | 1.17 | 0.007790 |
| ENSBTAG00000006368 | N4BP3 | 1.11 | 0.011447 |
| ENSBTAG00000047508 | ZNF580 | 1.10 | 0.009059 |
| ENSBTAG00000003675 | ADGRL1 | 1.09 | 0.003241 |
| ENSBTAG00000021338 | OAF | 1.08 | 0.041943 |
| ENSBTAG00000005934 | TTYH3 | 1.08 | 0.000463 |
| ENSBTAG00000038055 | KLF16 | 1.08 | 0.000217 |
| ENSBTAG00000015551 | RPL15P | 1.08 | 0.015028 |
| ENSBTAG00000019844 | ZNF467 | 1.07 | 0.000967 |
| ENSBTAG00000026505 | novel gene | 1.04 | 0.003884 |
| ENSBTAG00000019097 | CNTNAP1 | 1.03 | 0.041565 |
| ENSBTAG00000019718 | RPS15 | 1.02 | 0.003778 |
| ENSBTAG00000002550 | novel gene | 1.01 | 0.012842 |
| ENSBTAG00000018554 | novel gene | 1.01 | 0.007737 |
| ENSBTAG00000021755 | novel gene | 1.01 | 0.002418 |
| ENSBTAG00000019912 | CNTD1 | -1.00 | 0.004956 |
| ENSBTAG00000019612 | RNASE4 | -1.00 | 0.002928 |
| ENSBTAG00000009156 | CAMK2N1 | -1.04 | 0.045243 |
| ENSBTAG00000016415 | LTA4H | -1.06 | 0.000615 |
| ENSBTAG00000030434 | FUCA1 | -1.07 | 0.015667 |
| ENSBTAG00000009863 | BHLHE40 | -1.08 | 0.000001 |
| ENSBTAG00000021361 | SAMD3 | -1.14 | 0.018330 |
| ENSBTAG00000020467 | HRH4 | -1.18 | 0.018330 |
| ENSBTAG00000007490 | SULF2 | -1.19 | 0.007106 |
| ENSBTAG00000046323 | novel gene | -1.21 | 0.026782 |
| ENSBTAG00000038340 | CLNK | -1.28 | 0.008956 |
| ENSBTAG00000018446 | GCA | -1.29 | 0.003778 |
| ENSBTAG00000020602 | IDO1 | -1.31 | 0.005843 |
| ENSBTAG00000038141 | novel gene | -1.35 | 0.012842 |
| ENSBTAG00000019368 | IGFBP7 | -1.38 | 0.018655 |
| ENSBTAG00000003297 | OASL | -1.39 | 0.030394 |
| ENSBTAG00000004221 | ESM1 | -1.43 | 0.018655 |
| ENSBTAG00000009733 | FBP1 | -1.43 | 0.025667 |
| ENSBTAG00000018499 | ANXA8L1 | -1.46 | 0.006894 |
| ENSBTAG00000010728 | RAB44 | -1.46 | 0.025306 |
| ENSBTAG00000015177 | PRSS23 | -1.48 | 0.000296 |
| ENSBTAG00000015483 | CCR8 | -1.49 | 0.000161 |
| ENSBTAG00000018571 | IL1RL1 | -1.58 | 0.007106 |
| ENSBTAG00000004263 | ATP6V0A4 | -1.60 | 0.000018 |
| ENSBTAG00000010828 | LOC617313 | -1.77 | 0.006833 |
| ENSBTAG00000046158 | CFB | -1.78 | 0.027565 |
| ENSBTAG00000046693 | novel gene | -1.78 | 0.008086 |
| ENSBTAG00000013055 | novel gene | -1.85 | 0.001996 |
| ENSBTAG00000031265 | SVOPL | -1.85 | 0.005357 |
| ENSBTAG00000047632 | IGHE | -1.90 | 0.000040 |
| ENSBTAG00000020990 | P2RY14 | -1.91 | 0.003211 |
| ENSBTAG00000010057 | GZMB | -1.94 | 0.013539 |
| ENSBTAG00000007109 | ASB2 | -1.97 | 0.000198 |
| ENSBTAG00000038159 | novel gene | -1.97 | 0.011841 |
| ENSBTAG00000047449 | LOC100300483 | -1.98 | 0.000253 |
| ENSBTAG00000001125 | ADAMDEC1 | -2.00 | 0.018330 |
| ENSBTAG00000047816 | novel gene | -2.10 | 0.000217 |
| ENSBTAG00000038080 | LOC508858 | -2.11 | 0.001240 |
| ENSBTAG00000032051 | LOC100300896 | -2.14 | 0.009767 |
| ENSBTAG00000001292 | LTF | -2.34 | 0.002418 |
| ENSBTAG00000002092 | PI16 | -2.36 | 0.014950 |
