# Supplementary material for: Responses of Bovine Innate Immunity to Mycobacterium avium subsp. paratuberculosis Infection Revealed by Changes in Gene Expression and Levels of MicroRNA
Source: PLoS One. 2016 Oct 19;11(10):e0164461. doi: 10.1371/journal.pone.0164461 (PMC5070780; doi:10.1371/journal.pone.0164461)
Supplement: S3 Table — Genes with an FDR<0,05 and a Log2 fold change (Log2FC) <-1 or >1 were considered as differentially expressed. (DOCX) [file pone.0164461.s003.docx]

**S3 Table**. D**ifferentially expressed genes in the positive subject when compared with the exposed group.** Genes with an FDR<0,05 and a Log2 fold change (Log2FC) <-1 or >1 were considered as differentially expressed.

| Sequence ID | Gene symbol | LogFC | FDR |
| --- | --- | --- | --- |
| ENSBTAG00000047632 | IGHE | 1.71 | 0.006138 |
| ENSBTAG00000039555 | LOC101902937 | -2.69 | 0.036802 |
