# Supplementary material for: Responses of Bovine Innate Immunity to Mycobacterium avium subsp. paratuberculosis Infection Revealed by Changes in Gene Expression and Levels of MicroRNA
Source: PLoS One. 2016 Oct 19;11(10):e0164461. doi: 10.1371/journal.pone.0164461 (PMC5070780; doi:10.1371/journal.pone.0164461)
Supplement: S4 Table — (DOCX) [file pone.0164461.s004.docx]

| **miRNA** | **Target gene** | **Gene description** | **Regulation** | **Comparison** | **Effect** |
| --- | --- | --- | --- | --- | --- |
| bta-mir-1271 | TTYH3 | tweety family member 3 | upregulated | PP vs NN | increase the activity of phagocytes |
| bta-mir-19b | HIC1 | hypermethylated in cancer 1 | upregulated | PP vs NN | regulation of cytokine secretion |
| bta-mir-19b | IMPDH1 | IMP (inosine 5'-monophosphate) dehydrogenase 1 | upregulated | PP vs NN | activation of macrophages |
| bta-mir-19b-2 | HIC1 | hypermethylated in cancer 1 | upregulated | PP vs NN | regulation of cytokine secretion |
| bta-mir-19b-2 | IMPDH1 | IMP (inosine 5'-monophosphate) dehydrogenase 1 | upregulated | PP vs NN | activation of macrophages |
| Novel:14_7917 | ARL2 | ADP-ribosylation factor-like 2 | upregulated | PP vs NN | increase phagocytosis |
| Novel:14_7917 | AP2A1 | adaptor-related protein complex 2, alpha 1 subunit | upregulated | PP vs NN | increase phagocytosis |
| bta-mir-1271 | TTYH3 | tweety family member 3 | upregulated | NP vs NN | increase the activity of phagocytes |
| bta-mir-19b | ZBTB4 | zinc finger and BTB domain containing 4 | upregulated | NP vs NN | response of infected macrophages |
| bta-mir-19b-2 | ZBTB4 | zinc finger and BTB domain containing 4 | upregulated | NP vs NN | response of infected macrophages |

**S4 Table. Effects of gene regulation by miRNAs on the immune functions.**
